# Development of Thai-Specific Gestational Weight Gain Targets Using Asian BMI Cut-Points: Implications for Nursing and Midwifery Practice in Preventing Gestational Diabetes Mellitus

**DOI:** 10.3390/nursrep16080274

**Published:** 2026-08-04

**Authors:** Piyanut Xuto, Daniel Bressington, Lawitra Khiaokham, Patompong Khaw-on, Nantarat Matayaboon, Suraphan Sangsawang, Nantawan Janta, Pradab Sungplipan, Suratsawadee Wiangsuwan, Sujitra Chaiwatthanakorn, Ampaporn Phiwon, Kamonchanok Khetwang, Ratchaneewan Charuloedphong

**Affiliations:** 1 Faculty of Nursing, Chiang Mai University, 110/406 Intawaroros rd., Suthep, Muang, Chiang Mai 50200, Thailand; daniel.bressington@cmu.ac.th (D.B.); prueksalada.k@cmu.ac.th (L.K.); patompong.kh@cmu.ac.th (P.K.-o.); dolporn.m@cmu.ac.th (N.M.); 2 Regional Health Promotion Center 1, Department of Health, Ministry of Public Health, Chiang Mai 50100, Thailand; suraphan3107@gmail.com (S.S.); nium2907@gmail.com (N.J.); pradab.nso@gmail.com (P.S.); suratsawadee.wiang@gmail.com (S.W.); chaiwatthanakorn@gmail.com (S.C.); conference.152@gmail.com (A.P.); rn.lr.supernat@gmail.com (K.K.); rcharuloedphong@hotmail.com (R.C.)

**Keywords:** antenatal care, Asian populations, body mass index, gestational diabetes mellitus, gestational weight gain, midwifery, nursing

## Abstract

**Background**: Thai obstetric nurses follow WHO gestational weight gain (GWG) guidelines to prevent gestational diabetes mellitus (GDM). However, these Western-derived guidelines misclassify approximately 16% of Thai pregnant women, who carry excess metabolic risk at lower BMI values than Western populations. **Objectives**: To derive and validate Thai-specific GWG targets using Thai BMI cut-points across five BMI subgroups and assess implications for nursing practice in GDM prevention. **Methods**: Retrospective cohort study of 1396 Thai singleton pregnancies at a maternal and child hospital (2023–2025). GWG targets were derived as interquartile ranges (P25–P75) from a healthy-outcomes subgroup (*n* = 706), with validity confirmed by multivariable logistic regression and a 70:30 derivation/validation split. **Results**: GDM prevalence was 15.8%, with a rising crude trend that was attenuated after accounting for a mid-study change in screening protocol. Provisional Thai-specific GWG targets were underweight/normal weight 11.5–18.0 kg, overweight 9.7–18.0 kg, obesity class I 9.1–16.0 kg, and obesity class II+ 5.8–12.1 kg. Inadequate GWG in obesity class I was associated with a 3.92-fold GDM risk increase (aOR 3.92, 95% CI 2.12–7.24, *p* < 0.001). Adherence improved from 34.7% (IOM 2009) to 50.2% with Thai-specific targets. **Conclusions**: These Thai-specific GWG targets are provisional, single-centre pilot estimates. They appear clinically safe and are substantially more achievable than IOM 2009 criteria and are offered to inform BMI-appropriate antenatal counselling and to justify a multi-centre validation study, rather than as ranges that are ready for immediate national adoption.

## 1. Introduction

Gestational weight gain (GWG) is one of the most modifiable determinants of maternal and neonatal health. Insufficient weight gain during pregnancy is associated with low birth weight, preterm birth, and neonatal intensive care admission, while excessive GWG is linked to gestational diabetes mellitus (GDM), pre-eclampsia, macrosomia, and increased caesarean section rates [1,2]. The 2009 Institute of Medicine (IOM) guidelines remain the most widely cited international reference, recommending GWG ranges stratified by pre-pregnancy body mass index (BMI) [1]. These guidelines were predominantly derived from women of European descent and are not appropriate for Thai and other Asian populations, who exhibit distinct body composition and metabolic characteristics at lower BMI thresholds [3]. A systematic review and meta-analysis of over one million pregnancies across the USA, Western Europe, and East Asia [4] demonstrated that, when regional Asian BMI categories are applied, the proportion of Asian women classified as gaining above guidelines rises to levels comparable with Western populations, confirming that population-specific BMI classification fundamentally alters GWG risk assessment in Asian women [4].

A foundational challenge in applying IOM recommendations to Thai women lies in the BMI classification system. The IOM guidelines apply WHO cut points, defining overweight as BMI ≥ 25.0 kg/m^2^. However, the WHO Expert Consultation identified 23.0 kg/m^2^ as a public health action point for Asian populations, acknowledging that metabolic risk occurs at lower thresholds [5]. Contemporary evidence confirms that Asian adults develop insulin resistance, cardiovascular disease, and type 2 diabetes at substantially lower BMI values than Western populations, validating the lower cut-point as a biologically grounded public health threshold [5,6]. Consistent with this, Thai national clinical practice guidelines classify overweight as BMI ≥ 23.0 kg/m^2^ and obesity as BMI ≥ 25.0 kg/m^2^ [7]. Women with BMI 23.0–24.9 kg/m^2^ are therefore systematically misclassified as normal weight under IOM/WHO criteria despite carrying overweight-level obstetric risk. In the present study population, this group (16.0% of participants) had a GDM rate of 17.5% and a preeclampsia/gestational hypertension (PE/GHT) rate of 4.9%, substantially higher than true normal weight women (GDM 13.8%, PE 1.4%), underscoring the clinical consequences of this misclassification.

GDM is a critical and escalating concern in the Thai context. A 20-year trend analysis at our study site documented a dramatic increase in GDM prevalence from 3.4% in 2003 to 22.0% in 2022 (*p* < 0.001) [8], with the most recent annual institutional report documenting a further rise to 25% of all obstetric patients in 2025 [9]. Nationally, the prevalence of overweight and obesity among Thai adults increased from approximately 32% to over 43% between 2009 and 2020 [10], creating a compounding risk environment that existing GWG guidelines are ill-equipped to address. More recent national survey data indicate that overweight and obesity now affect over 50% of the Thai adult population [11], highlighting the accelerating trajectory of metabolic risk in women of reproductive age. Globally, the International Diabetes Federation estimates that approximately 14% of all pregnancies are affected by gestational diabetes, with Southeast Asia carrying the highest regional burden at 28.0%—the highest of any world region [12]. A scoping review of GDM in Southeast Asia confirmed that Thailand reports among the highest hospital-based GDM prevalence in the region, and that national surveillance systems remain inadequate [13]. Critically, GDM is not merely a pregnancy complication: women diagnosed with GDM face a sevenfold increased lifetime risk of type 2 diabetes, and their offspring face a two- to eightfold risk of obesity, metabolic syndrome, and type 2 diabetes in childhood and adulthood [14], creating an intergenerational cardiometabolic cycle that effective midwifery prevention—anchored to population-specific GWG guidance—has a unique opportunity to interrupt.

Two prior Thai studies derived population-specific GWG recommendations using retrospective cohort designs [15,16], thereby improving adherence over the IOM 2009 recommendations from approximately 35% to over 56%. However, both applied WHO BMI cut-points, misclassifying the overweight group as described above, and neither disaggregated obesity into class I (BMI 25.0–29.9 kg/m^2^) and class II+ (≥30.0 kg/m^2^), despite clinically important differences between these subgroups. The guideline gap extends beyond Thailand: a systematic appraisal of ten pregnancy weight gain clinical practice guidelines across the Asia-Pacific region found that 90% were rated as low quality under AGREE II criteria, with substantial variation in GWG recommendations and no regional consensus on Asian-specific BMI classification [17]. This evidence-based deficit has direct clinical consequences: when IOM guidelines are applied to Asian women using WHO BMI categories, a large proportion are misclassified as gaining within or below recommended ranges despite showing elevated obstetric risk, as demonstrated by a meta-analysis of over one million women that applied regional BMI categories specifically to Asian subgroups [4].

Nurses and midwives are the primary providers of antenatal care in Thailand and are uniquely positioned to counsel women on GWG at every visit. Evidence consistently shows that women whose midwives discuss GWG targets are more likely to gain weight within recommended ranges [18,19]. Yet a systematic review found that midwives frequently reported insufficient knowledge and lack of population-specific guidance as barriers to effective weight management counselling [20]. A national survey of Thai nurse-midwives found that weight management services were absent in most tertiary care units and that the majority of overweight and obese pregnant women had not received individualised GWG counselling [21]. A mixed-methods systematic review of barriers and enablers to GWG counselling across maternity settings identified the absence of population-specific guidelines, insufficient provider training, and lack of institutional weight monitoring protocols as the primary structural impediments to routine practice [22]. The absence of a Thai population-specific, BMI-classification-congruent GWG guideline represents a critical gap in the evidence base for midwifery practice.

The present study addresses this gap by deriving and internally validating Thai-specific GWG targets using Thai BMI cut-points across five BMI subgroups, assessing their association with clinically important pregnancy outcomes, and evaluating their implications for midwifery-led antenatal care in the context of rising GDM burden.

## 2. Materials and Methods

### 2.1. Study Design and Setting

This retrospective cohort study was conducted at the Regional Health Promotion Center 1, Department of Health, Ministry of Public Health, Chiang Mai, Thailand, which is a specialized hospital with a 60-bed capacity. Antenatal care is delivered primarily by nurses and midwives, supported by obstetricians. Electronic antenatal records from three consecutive annual cohorts were extracted, covering January 2023–December 2025. The study was conducted and reported on 18 March to 20 April 2026, in accordance with the Strengthening the Reporting of Observational Studies in Epidemiology (STROBE) guidelines [23]. Ethical approval was granted by the Institutional Review Board under Exemption Category 5 (secondary analysis of existing electronic medical records with no direct participant contact). The secondary data collection was approved by the Institutional Review Board of the Faculty of Nursing, Chiang Mai University (Code: 2026-EXEM002, approved 11 March 2026) and the Regional Health Promotion Center 1, Department of Health, Ministry of Public Health, Chiang Mai, Thailand (Code: 07/2569, approved 27 February 2026), covering analysis of existing electronic medical records with no direct participant contact. Individual consent was waived by the ethics committee in accordance with national regulations for secondary analysis of routinely collected health data.

### 2.2. Participants

Inclusion criteria were: Thai nationality; singleton pregnancy; first antenatal care visit at ≤14 weeks of gestation; age 18–45 years; height >100 cm; available pre-pregnancy weight and delivery weight; gestational age ≥ 37 weeks at delivery (except for preterm outcome analysis); live singleton birth; and absence of documented fetal anomaly. Women were excluded if they had pre-existing diabetes mellitus (ICD-10: O24.1, O24.2, O24.3), multiple gestation (O30x), or pre-existing hypertension (O10x). Cases with implausible total GWG values (<−5 kg or >40 kg) were excluded as likely data entry errors. After sequential application of all exclusion criteria, 1396 Thai women were included in the final analysis.

### 2.3. BMI Classification

Pre-pregnancy BMI was calculated as weight recorded at the first antenatal visit (≤14 weeks) divided by height squared (kg/m^2^). BMI was classified using Thai-specific cut-points into five groups: underweight (<18.5 kg/m^2^), normal weight (18.5–22.9 kg/m^2^), overweight (23.0–24.9 kg/m^2^), obesity class I (25.0–29.9 kg/m^2^), and obesity class II+ (≥30.0 kg/m^2^), consistent with Thai national clinical practice guidelines and WHO Asia-Pacific recommendations [5,7]. This five-group classification was determined a priori and validated empirically by significant differences in GWG medians (Kruskal–Wallis, *p* < 0.001) and PE/GHT rates between obesity subgroups (class I 3.0% vs. class II+ 16.1%; χ^2^ = 31.4, *p* < 0.001).

### 2.4. Gestational Weight Gain Measurement

Total GWG was calculated as the difference between delivery weight and pre-pregnancy weight (kg). Delivery weight was recorded at the most recent antenatal visit preceding delivery or on the delivery day itself.

### 2.5. GDM Ascertainment

GDM was ascertained through a two-phase process reflecting a documented change in hospital screening protocol. In Years 2023 and 2024, the hospital employed selective screening using a 50 g glucose challenge test (GCT) at 24–28 weeks of gestation, with a 100 g oral glucose tolerance test (OGTT) administered to women with GCT ≥ 140 mg/dL. In 2025, the protocol was updated to universal OGTT screening at the first antenatal visit. For Years 2023 and 2024, GDM status was verified by a trained nursing team through direct electronic medical record review (*n* = 165 cases reviewed), using ICD-10 codes O24.0 and O24.4 as primary ascertainment codes. For 2025, GDM was ascertained directly from O24.4x. In total, 220 GDM cases (15.8%) were identified. Because a change in screening strategy can itself generate an apparent secular rise in prevalence, screening protocol (selective versus universal) was carried forward as a covariate into pre-specified sensitivity analyses of the annual GDM trend (Section 2.10), and the residual impact of the change is additionally addressed as a study limitation.

### 2.6. Derivation of GWG Targets

GWG targets were derived using a healthy outcomes methodology consistent with prior Thai work [15]. The healthy outcomes subgroup was defined as women with verified absence of all the following: GDM, PE/GHT (ICD-10: O11, O13, O14), caesarean section, low birth weight (<2500 g), macrosomia (≥4000 g), and preterm birth (<37 weeks), who delivered at 37–42 weeks of gestation. The 25th and 75th percentiles (IQR) of GWG within each BMI subgroup of the healthy outcomes population were used as the lower and upper bounds of the recommended GWG range.

### 2.7. Extended Zone Safety Analysis: Analytical Approach

Because the Thai-specific upper bounds exceed IOM 2009 recommendations for the overweight, obesity class I, and obesity class II+ groups, an explicit safety analysis was conducted for the “extended zone”: women whose GWG exceeded the IOM upper bound but remained within the Thai-specific P75. Rates of all six adverse outcomes were compared between the extended zone and the IOM-adequate zone using chi-square tests and multivariable logistic regression adjusted for maternal age and nulliparity.

### 2.8. Statistical Analysis

Descriptive statistics are reported as means ± standard deviations for continuous variables and as frequencies with percentages for categorical variables. Normality was assessed using the Kolmogorov–Smirnov test for subgroups > 50 and the Shapiro–Wilk test for subgroups ≤ 50. GWG distributions were non-normal in all BMI groups (*p* < 0.05), justifying use of the Kruskal–Wallis test for between-group comparisons. Chi-square tests were used for categorical outcome comparisons. Multivariable logistic regression models estimated adjusted odds ratios (aOR) for each clinical outcome (GDM, caesarean section, PE/GHT, low birth weight, macrosomia, preterm birth) by GWG category (inadequate or excessive, each referenced to adequate GWG), adjusted for maternal age and nulliparity as a priori confounders, within each BMI subgroup. Prior to multivariable logistic regression modelling, assumptions were verified for each subgroup model: multicollinearity was acceptable (VIF < 2.0 for all predictors); linearity of maternal age with log-odds was confirmed by the Box-Tidwell test (all *p* > 0.05); no influential observations were identified (Cook’s distance < 1.0 in all models; maximum = 0.136); and the events-per-variable criterion of ≥10 was met for all models reporting significant findings.

Adequate GWG was defined as GWG within the Thai-specific P25–P75 range. Internal validation was performed by randomly splitting the dataset 70:30 (derivation set n = 977, validation set *n* = 419) using stratified sampling by BMI group (random seed = 42). GWG targets were re-derived from the derivation set and regression findings were assessed for directional consistency in the validation set. A two-tailed significance threshold of *p* < 0.05 was applied throughout. All analyses were performed using Python 3.12 (Python Software Foundation, Wilmington, DE, USA) on Google Colaboratory (Google LLC, Mountain View, CA, USA; https://colab.research.google.com) with the statsmodels 0.14 and SciPy 1.11 packages [24].

### 2.9. Derivation of Provisional Clinical Guidance Ranges

The empirically derived P25–P75 targets were translated into provisional clinical guidance ranges using five decision rules, applied in the order below so that the proposed ranges are fully reproducible and auditable. Rule 1 (empirical admissibility): no proposed bound was permitted to fall outside the observed GWG distribution of the healthy-outcomes subgroup for that BMI group. Rule 2 (deference where external sources classify the same women): for the underweight and normal weight groups, where IOM 2009 [1], Siriraj 2014 [15] and the present study classify an identical population, the lower bound was set at the arithmetic mean of the three lower bounds rounded to the nearest whole kilogram, and the upper bound at the most conservative (lowest) of the three upper bounds. Rule 3 (upper ceiling): for all non-underweight groups, the proposed upper bound was capped at 16 kg, the highest upper limit endorsed by any established source for a non-underweight population, even where our empirical P75 was higher. Rule 4 (lower-bound rounding): empirically derived lower bounds were rounded down to the nearest whole kilogram; where the fractional part was less than 0.5 kg, a further 1 kg was subtracted so that the whole-kilogram figure counselled to women never sits immediately above the empirical 25th percentile. This deliberately permissive treatment of the lower bound reflects both measurement uncertainty in pre-pregnancy weight, which was taken at ≤14 weeks, and our finding that inadequate gain carries substantive risk. Rule 5 (small-subgroup deference): where the healthy outcomes subgroup contained fewer than 50 women, the lower bound was taken from IOM 2009 rather than from the imprecise empirical P25, and the empirical P75 was rounded down to the nearest whole kilogram; this rule was only applied to obesity class II+.

### 2.10. Sensitivity and Supplementary Analyses

Three further analyses were specified to address ascertainment change, the shape of the exposure–outcome relationship, and nursing process confounding. First, the annual GDM trend was re-examined using three complementary approaches: (a) a multivariable logistic regression model with GDM as the outcome and calendar year, screening protocol (selective versus universal), maternal age, BMI group and nulliparity as covariates; (b) a restricted trend analysis confined to the two selective-screening years (2023 and 2024), during which ascertainment was constant. Because the calendar year and screening protocol are partially collinear by design, approach (b), rather than approach (a), is treated as the primary evidence for whether the rise is real. Second, to describe the shape and not only the direction of the GWG–outcome relationship, restricted cubic spline logistic regression models were fitted with total GWG entered as a continuous exposure (three knots placed at the 10th, 50th and 90th percentiles of the GWG distribution), adjusted for maternal age and nulliparity and stratified by BMI group, GDM, PE/GHT, and low birth weight. Departure from linearity was tested by likelihood-ratio comparison with the corresponding linear model, and adjusted predicted probabilities with 95% confidence bands were plotted against GWG with the derived P25 and P75 overlaid.

## 3. Results

### 3.1. Sample Characteristics

A total of 1396 Thai women were included in the final analysis (Year 2023: n = 528, 37.8%; Year 2024: n = 475, 34.0%; Year 2025: n = 393, 28.2%). Mean maternal age was 29.5 ± 5.2 years; 54.6% were nulliparous. Mean pre-pregnancy BMI was 22.9 ± 4.2 kg/m^2^ and mean total GWG was 13.8 ± 5.7 kg. Under Thai-specific BMI classification, 169 women (12.1%) were underweight, 644 (46.1%) normal weight, 223 (16.0%) overweight, 267 (19.1%) obesity class I, and 93 (6.7%) obesity class II+. Baseline characteristics by BMI group are presented in Table 1.

Overall GDM prevalence was 15.8% (*n* = 220), with a significant rising trend: 13.3% in Year 2023, 15.6% in Year 2024, and 19.3% in Year 2025 (χ^2^ trend = 7.82, *p* = 0.005). GDM rates increased with BMI category from 11.2% in underweight to 26.9% in obesity class II+ women. The PE/GHT rate was markedly higher in obesity class II+ women (16.1%) compared to obesity class I (3.0%; *p* < 0.001), providing empirical justification for the five-group classification. Because the screening protocol changed between 2024 and 2025, this crude trend was re-examined under constant ascertainment. Restricted to the two selective-screening years, GDM prevalence did not differ significantly (13.3% in 2023 versus 15.6% in 2024; χ^2^ = 1.10, *p* = 0.295), indicating that the significant overall trend is driven principally by the single year in which universal screening was introduced. In the multivariable model, the association of calendar year with GDM was aOR 1.25 (95% CI 0.87–1.80, *p* = 0.234) after adjustment for screening protocol; aOR 0.97 (95% CI 0.52–1.82, *p* = 0.923) after adjustment for screening protocol, maternal age, BMI group, and nulliparity (Table S1). These analyses indicate that a substantial proportion of the apparent rise reflects intensified case ascertainment rather than a true increase in incidence.

### 3.2. Thai-Specific GWG Targets

**Figure 1:**
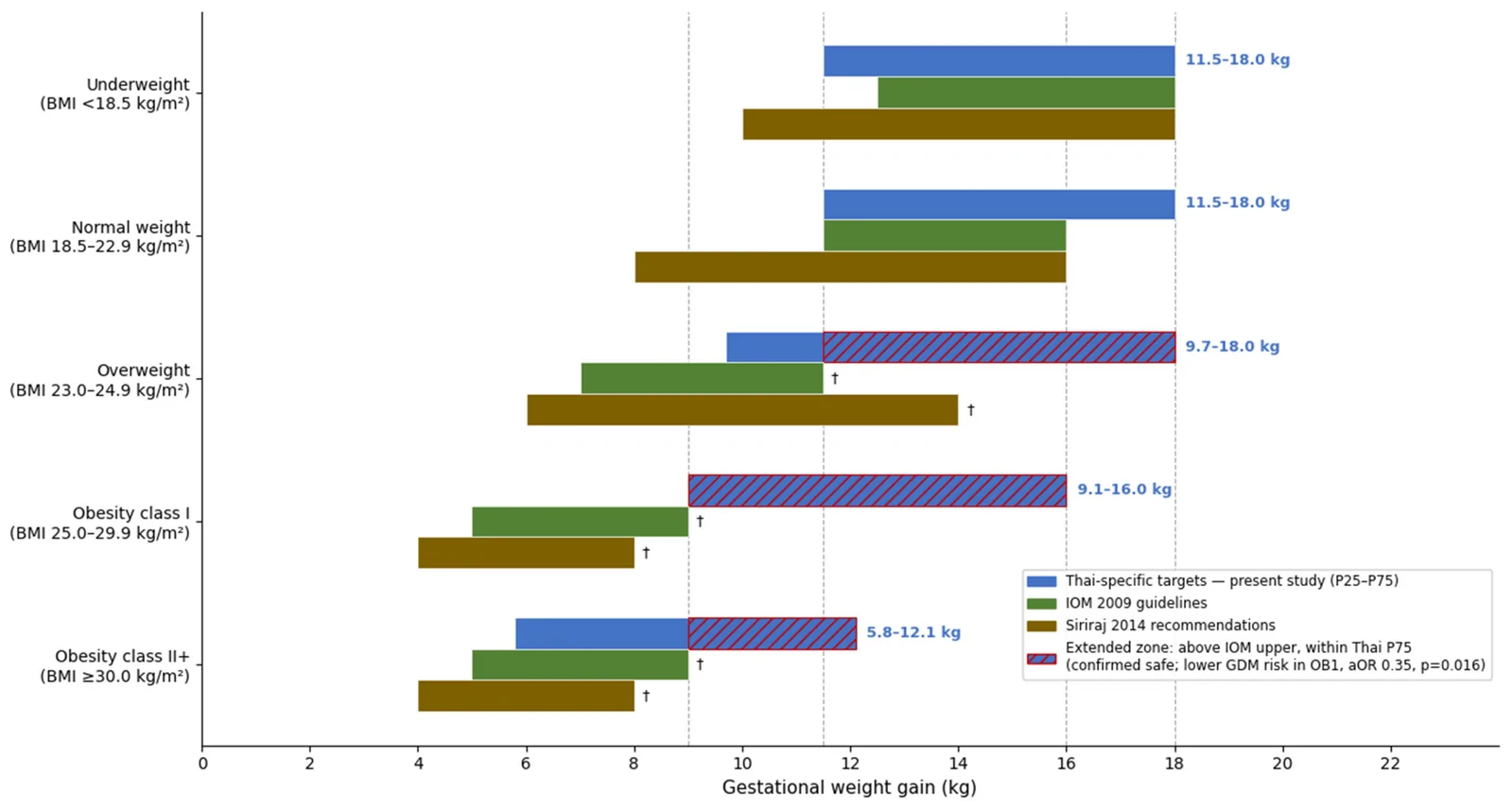
Gestational weight gain range by pregnancy–BMI group: Thai-specific targets (present study) vs. IOM (2009) and Siriraj (2024). Note: † Thai-specific target upper bound exceeds IOM/Siriraj for this group. Dashed vertical lines indicate the IOM 2009 upper bound thresholds for reference.

The healthy outcomes subgroup comprised 706 women (50.6%): underweight *n* = 109, normal weight *n* = 362, overweight *n* = 101, obesity class I *n* = 107, and obesity class II+ *n* = 27. Thai-specific GWG targets, derived as P25–P75 from this subgroup, are presented in Table 2, alongside IOM 2009 and Siriraj 2014 recommendations. Figure 1 provides a visual comparison of GWG ranges across all three sources by BMI group. Thai-specific upper bounds were substantially higher than IOM 2009 for the overweight, obesity class I, and obesity class II+ groups, reflecting the observed GWG distributions of Thai women who achieved healthy pregnancy outcomes. The obesity class I target (9.1–16.0 kg) is substantially broader than the IOM 2009 class (5.0–9.0 kg), reflecting clinically important heterogeneity among obesity subgroups that is obscured by the four-group WHO classification.

### 3.3. GWG Classification and Adherence

Table 3 compares GWG classification under three criteria systems applied to the same 1396 women. Under IOM 2009 guidelines, 34.7% of women (*n* = 484) achieved adequate GWG; 39.5% (*n* = 551) exceeded the upper bound. Under Thai-specific criteria, adherence increased to 50.2% (*n* = 701), while the proportion classified as excessive decreased to 23.6% (*n* = 329)—a 40% relative decrease in excessive GWG labelling. Critically, outcome rates among women classified as adequate did not differ significantly between criteria systems: GDM 15.3% (IOM adequate) vs. 14.7% (Thai adequate; *p* = 0.841), PE/GHT 2.1% vs. 2.3% (*p* = 0.962), and LBW 3.9% vs. 4.1% (*p* = 0.975), confirming that the broader Thai-specific adequacy classification does not confer additional risk.

### 3.4. Multivariable Logistic Regression

Significant associations between GWG category and adverse outcomes are presented in Table 4. Among obesity class I women (BMI 25.0–29.9 kg/m^2^), inadequate GWG was associated with a nearly fourfold increase in GDM risk (aOR 3.92, 95% CI 2.12–7.24, *p* < 0.001). Among overweight women (BMI 23.0–24.9 kg/m^2^), GWG exceeding the Thai-specific upper bound of 18.0 kg was associated with a 7.57-fold increase in PE/GHT risk (aOR 7.57, 95% CI 1.51–37.86, *p* = 0.014); this “excessive” category refers specifically to women gaining above the Thai-specific P75 of 18.0 kg, not within the extended zone of 11.5–18.0 kg (see Section 3.5). Among normal weight women (BMI 18.5–22.9 kg/m^2^), inadequate GWG was significantly associated with increased low birth weight risk (aOR 3.43, 95% CI 1.46–8.08, *p* = 0.005) and preterm birth risk (aOR 2.83, 95% CI 1.20–6.63, *p* = 0.017). The mechanistic interpretation of these findings is presented in Section 4.2.

### 3.5. Extended Zone Safety Analysis

The extended zone was defined as GWG above the IOM 2009 upper bound but within the Thai-specific P75 for each BMI group. In no BMI group did women in the extended zone have significantly higher rates of GDM, PE/GHT, LBW, macrosomia, or preterm birth compared with women in the IOM-adequate zone (all *p* > 0.05). In obesity class I, women in the extended zone (GWG 9.0–16.0 kg; *n* = 113) had a GDM rate of 11.5% compared with 28.3% in the IOM-adequate zone (GWG 5.0–9.0 kg; *n* = 53; aOR 0.35, 95% CI 0.15–0.82, *p* = 0.016), indicating significantly lower GDM risk in the extended zone than within the IOM-recommended range. For overweight women, the extended zone (GWG 11.5–18.0 kg; *n* = 90) showed no significant increase in PE/GHT or any other adverse outcome compared with the IOM-adequate zone. The mechanistic explanation for these findings, including reconciliation with the regression findings, is presented in Section 4.2.

### 3.6. Internal Validation

GWG targets re-derived from the derivation set (*n* = 977) differed from full-dataset targets by less than 1.0 kg in four of five BMI groups; obesity class I targets were identical (9.1–16.0 kg). The 1.3 kg difference in overweight P25 (derivation: 11.0 kg vs. full: 9.7 kg) reflects the smaller healthy overweight subgroup in the derivation set (*n* = 70 vs. n = 101 overall) and is unlikely to be clinically significant. All eight logistic regression findings that could be assessed in both sets showed the same OR direction in the validation set. Outcome rates among adequate women in the validation set (GDM 14.4%, PE 1.4%, LBW 2.9%) were closely consistent with full-dataset rates (GDM 14.7%, PE 2.3%, LBW 4.1%), supporting the stability of the derived targets.

### 3.7. Dose–Response Relationship Between GWG and Adverse Outcomes

**Figure 2:**
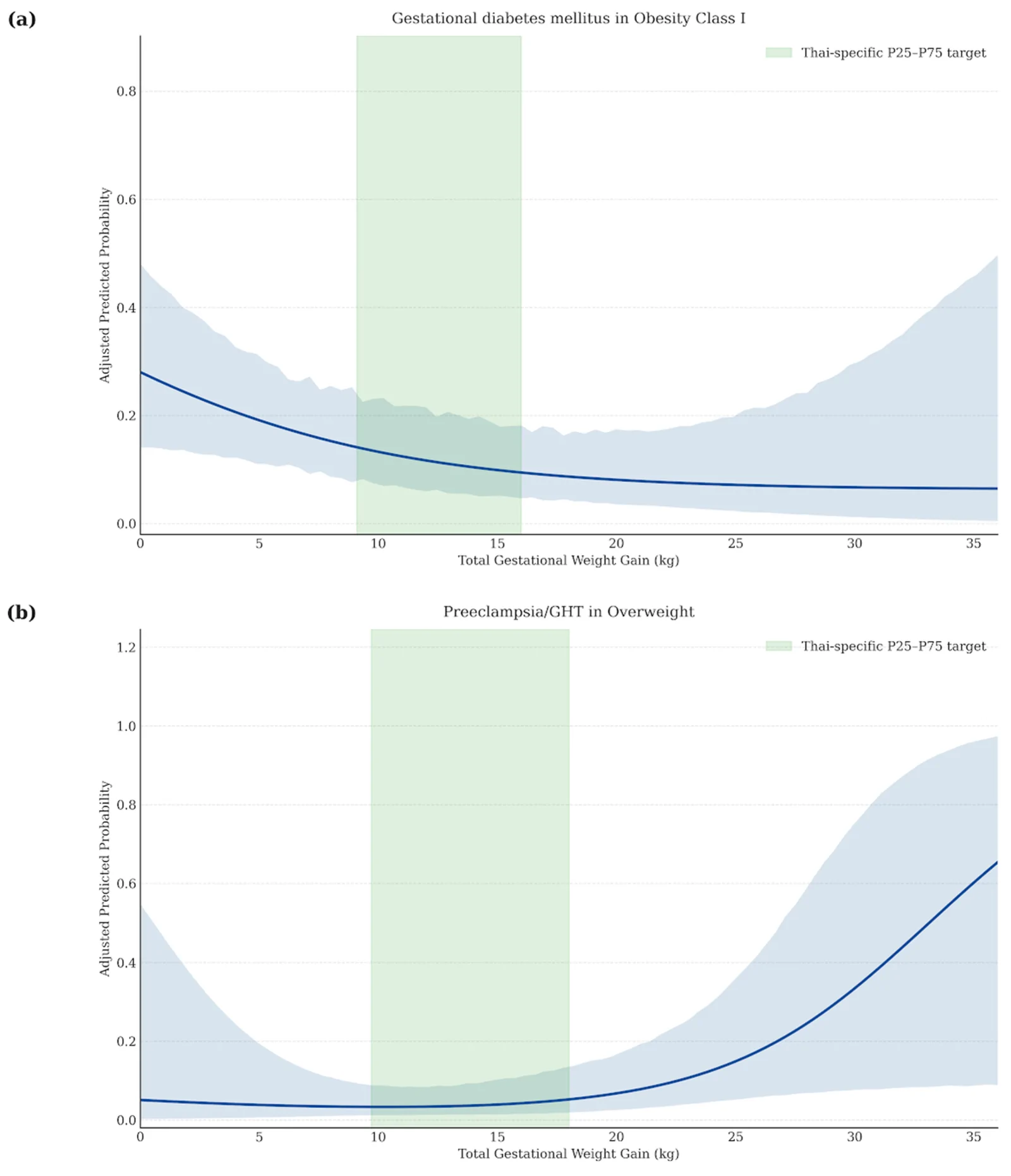

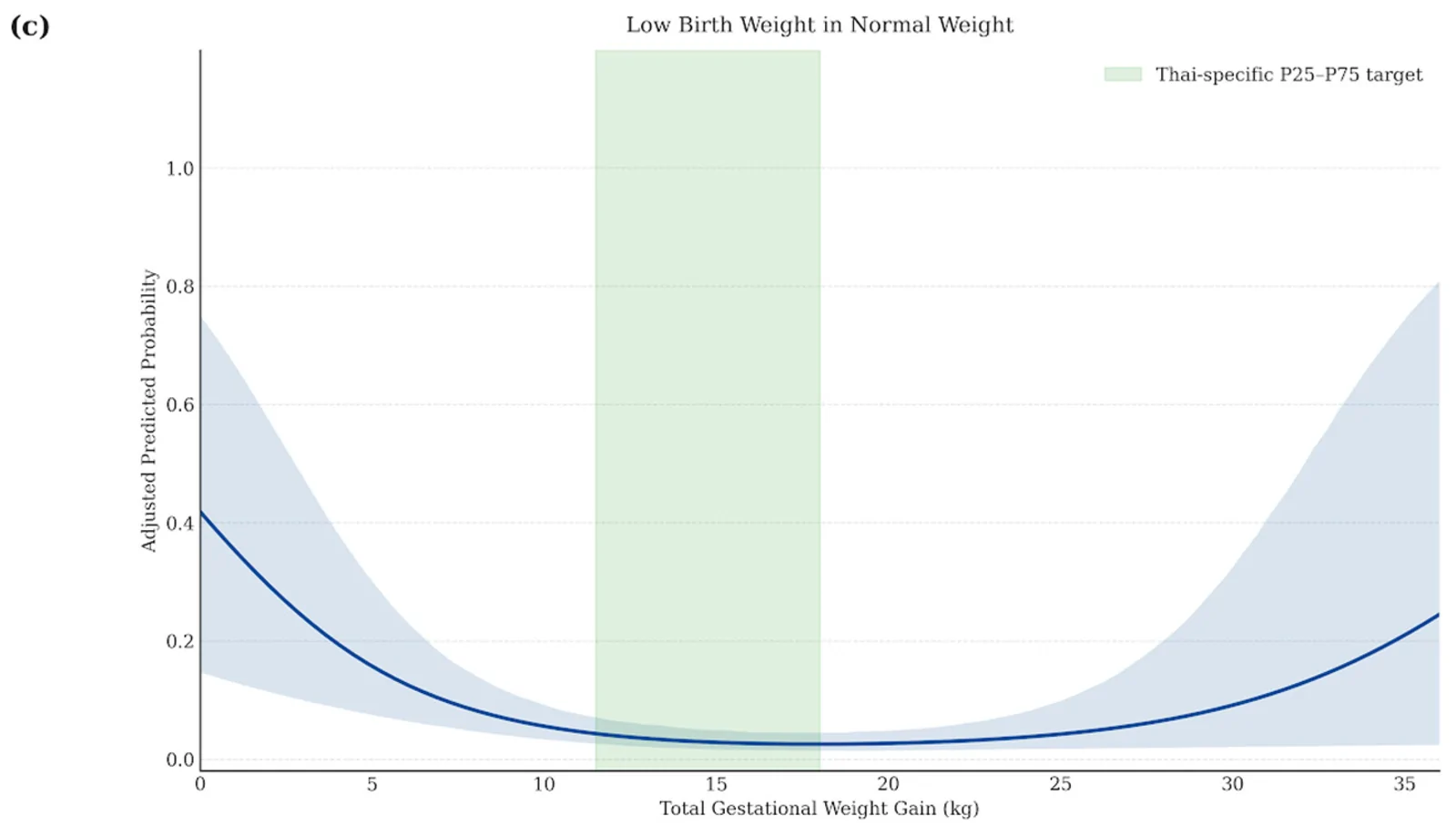
Dose–response relationship between total gestational weight gain (GWG) and adverse pregnancy outcomes, modelled by restricted cubic splines. Adjusted predicted probabilities (solid blue lines adjusted for maternal age and nulliparity) with 95% confidence bands (shaded blue areas) for: (**a**) gestational diabetes mellitus (GDM) in women with obesity class I; (**b**) preeclampsia or gestational hypertension (PE/GHT) in women with overweight; and (**c**) low birth weight (LBW) in women with normal weight. The green shaded area represents the provisional Thai-specific GWG target (P25–P75) derived for each BMI group.

Restricted cubic spline modelling was specifically applied to the primary adverse outcomes that demonstrated significant associations in the prior multivariable logistic regression, including GDM in obesity class I, PE/GHT in overweight, and LBW in normal weight. We refrained from fitting spline models for outcomes with very low event rates, such as macrosomia (*n* = 35 overall, 2.5%), to avoid model overfitting and statistically unstable estimates. For gestational diabetes mellitus (GDM), the adjusted probability curve in women with obesity class I exhibited an L-shaped pattern, characterised by a monotonic decline that flattened at higher levels of gestational weight gain (GWG), with no evidence of non-linearity (likelihood-ratio test, *p* = 0.464) (Figure 2a). In women with overweight, the curve for preeclampsia/gestational hypertension (PE/GHT) was clearly U-shaped; predicted risk was minimised at approximately 10.3 kg, which is within the provisional Thai-specific target range, before increasing sharply beyond 15.0 kg (Figure 2b). A similar U-shaped association was observed for low birth weight among normal weight women (*p* = 0.006 for non-linearity), with risk declining across the lower end of the GWG distribution to a minimum at approximately 18.0 kg and then rising again at higher weight gains (Figure 2c). Notably, the GWG values associated with the lowest predicted risk were contained within the derived P25–P75 target ranges in two of the three BMI groups examined (PE/GHT and LBW), thereby providing continuous-exposure support for the categorically derived targets reported above.

## 4. Discussion

### 4.1. Thai-Specific Targets: Correcting Systematic BMI Misclassification

The central contribution of this study is the derivation of GWG targets beginning from the correct BMI classification for Thai women. By applying Thai-specific cut-points (overweight ≥ 23.0 kg/m^2^), we correctly classified 16% of our study population who were previously misidentified as normal weight under WHO criteria. Women with BMI 23.0–24.9 kg/m^2^ had a GDM rate of 17.5% and a PE rate of 4.9%—substantially higher than true normal weight women (GDM 13.8%, PE 1.4%)—consistent with the WHO Expert Consultation’s identification of BMI 23.0 kg/m^2^ as a public health action point for Asian populations [5]. Counselling these women using normal weight GWG targets, therefore, not only misrepresents their actual risk, but may actively encourage weight gain in an already at-risk group. Our five-group classification extends prior Thai work by separating obesity into class I and class II+, justified by significant differences in median GWG (12.0 vs. 8.7 kg; *p* < 0.001) and PE rates (3.0% vs. 16.1%; *p* < 0.001) between subgroups.

### 4.2. Implications for Nursing and Midwifery Practice: Pathophysiological Basis and Clinical Translation

The escalating GDM prevalence at our study site—from 13.3% in 2023 to 19.3% in 2025 and reaching 25% of all obstetric patients in the most recent institutional annual report [9]—represents a public health emergency that midwives are uniquely positioned to address. This trajectory mirrors the broader northern Thailand trend documented over two decades [8]. Given that GWG is among the few pregnancy-related risk factors directly modifiable through antenatal counselling and that midwives conduct the majority of antenatal visits in Thailand, translation of evidence-based, population-specific GWG targets into midwifery practice is a critical and urgent prevention strategy. Beyond the immediate pregnancy, the intergenerational stakes are substantial: GDM confers a sevenfold increased lifetime risk of type 2 diabetes in the mother and a two- to eightfold increased cardiometabolic risk in the offspring [14], perpetuating the very obesity epidemic that drives GDM prevalence. Midwifery-led GWG prevention therefore operates as a primary intervention at the most upstream point in this intergenerational cycle.

The finding that inadequate GWG in obesity class I women is associated with a 3.92-fold increase in GDM risk (Section 3.4) requires careful pathophysiological interpretation before it can be translated into midwifery guidance. The raw GDM event rate within this inadequate subgroup was 35.3%—substantially higher than in the adequate or excessive subgroups—which appears paradoxical, as one might expect excessive rather than insufficient weight gain to drive GDM risk. However, this pattern reflects a well-recognized pathomechanism: women with pre-obesity who develop GDM commonly have pre-existing, pregnancy-independent insulin resistance that precedes conception. During pregnancy, this underlying insulin resistance impairs the normal placental and adipose tissue adaptations that drive gestational weight accretion, thereby simultaneously constraining weight gain capacity and elevating glucose intolerance risk through progressive beta-cell insufficiency [25]. Inadequate GWG in obesity class I women is therefore not a cause of GDM, but rather a clinical marker of the most severe metabolic phenotype within this group—women whose beta-cell reserve is already insufficient to compensate for the physiological insulin resistance of pregnancy. The critical midwifery implication is that counselling obese women to restrict weight gain, which remains common practice in many Thai antenatal units [21], is not only unlikely to prevent GDM, but may paradoxically worsen maternal nutritional status without addressing the underlying metabolic dysfunction. Midwives should instead support all obesity class I women in achieving GWG within the evidence-based target range of 8–16 kg and recognize that failure to achieve this range is itself a clinical signal warranting closer metabolic surveillance.

The 7.57-fold increase in PE/GHT risk associated with GWG exceeding 18.0 kg in overweight women is consistent with established pathophysiology linking excess gestational adipose accumulation to systemic inflammation, endothelial dysfunction, and placental insufficiency [26]. Thai-classified overweight women already carry elevated baseline vascular risk, making this group particularly sensitive to weight gain beyond 18.0 kg. Nurses and midwives should intensify blood pressure monitoring in overweight women whose GWG trajectory exceeds this threshold [7].

Among normal weight women, the association between inadequate GWG and low birth weight (aOR 3.43, 95% CI 1.46–8.08, *p* = 0.005) is consistent with a large individual participant data meta-analysis of 93,337 pregnancies across 48 studies from low- and middle-income countries, which found severely inadequate GWG associated with a 62% higher risk of low birth weight (RR 1.62, 95% CI 1.51–1.72) [27]. The association between inadequate GWG and preterm birth (aOR 2.83, 95% CI 1.20–6.63, *p* = 0.017) is corroborated by two independent large-scale syntheses: a JAMA meta-analysis found that GWG below IOM recommendations was associated with a 70% higher risk of preterm birth (OR 1.70, 95% CI 1.32–2.20) [2], and an individual participant data analysis of 36 randomized trials across 16 countries similarly found that inadequate GWG was associated with a near-twofold increase in preterm birth risk (aOR 1.94, 95% CI 1.31–2.28) [28]. Concurrently, chronic maternal energy deficit activates the fetal hypothalamic–pituitary–adrenal stress axis, elevating fetal cortisol and prostaglandin synthesis, which shortens gestational length and increases preterm birth risk [29]. These findings underscore that the lower bound of the Thai-specific GWG target is clinically as important as the upper limit, and that midwifery counselling must address the risk of insufficient as well as excessive weight gain—particularly for the 46% of women in this cohort who were normal weight.

The extended zone analysis (Section 3.5) and the regression finding for overweight women (Section 3.4) are consistent, not contradictory: “excessive” GWG in Section 3.4 refers to gains above the Thai-specific P75 of 18.0 kg, whereas the extended zone spans 11.5–18.0 kg—entirely below that ceiling. Together, they delineate a precise safe operating range: gains up to 18.0 kg carry no measurable vascular risk; gains beyond 18.0 kg carry substantial risk. The same logic confirms that restricting obesity class I women to the IOM-adequate zone (5–9 kg) is itself risk-conferring, as the threefold lower GDM rate in the 9–16 kg extended zone demonstrates. Within this single cohort, therefore, the proposed ranges were not associated with excess risk relative to the IOM-adequate zone; confirmation in independent populations remains necessary before they are applied as fixed clinical thresholds.

A persistent barrier to translating GWG evidence into Thai midwifery practice has been the absence of population-specific, BMI-classification-congruent targets that practitioners can apply with confidence. A national survey documented that midwives routinely used inconsistent GWG criteria and that most overweight and obese pregnant women had not received individualized GWG counselling [21]. Evidence from other settings indicates that midwife-led weight monitoring using structured GWG charts combined with brief, goal-directed counselling is both feasible and effective in improving adherence to routine antenatal care [19,30]; in the Asian context specifically, a nurse-led mobile health intervention in Taiwan significantly reduced excessive GWG in overweight and obese women, demonstrating the feasibility of technology-supported, nurse-delivered GWG management aligned with population-specific targets [31]. The present study directly addresses this evidence gap by providing validated, BMI-group-specific targets that are confirmed to be internally stable and clinically safe. Implementation should be supported by institutional policies mandating weight recording at each antenatal visit, provision of Thai BMI-classified GWG charts as standard antenatal materials, and inclusion of population-specific GWG counselling in pre-registration and continuing midwifery education curricula.

Two further associations in Table 4, although statistically significant, warrant brief comment because they run counter to intuitive expectation: caesarean section in normal weight women with inadequate GWG (aOR 0.57, 95% CI 0.36–0.90, *p* = 0.016) and gestational diabetes in overweight women with excessive GWG (aOR 0.29, 95% CI 0.09–0.88, *p* = 0.029). On the surface, less weight gain is associated with fewer caesarean sections and more weight gain with less GDM—the opposite of what the obesity class I and PE/GHT findings above would suggest. One plausible explanation is reverse temporal sequencing: women who deliver preterm or via early emergency caesarean section have a shorter window over which to accumulate GWG, which could mechanically lower their measured total GWG independent of any protective effect. Given the modest subgroup sizes involved, these two associations should be interpreted with caution and flagged for confirmation in future, prospectively designed studies. Beyond this interpretive caution, both estimates are statistically fragile. The overweight excessive-GWG subgroup contained 60 women with four GDM events, so the odds ratio of 0.29 rests on a very small number of events and its confidence interval spans an order of magnitude (0.09–0.88); this model falls below the events-per-variable criterion of ten applied elsewhere in our analysis, and the estimate should be regarded as unstable rather than informative. Neither association was pre-specified, neither was adjusted for gestational age at delivery, and neither survives a conservative correction for the number of outcomes–subgroup combinations tested. We therefore report them as exploratory observations only. They are not used in deriving, defending, or interpreting the proposed GWG ranges, and they should not be read as evidence that lower weight gain protects against caesarean section or that higher weight gain protects against GDM.

### 4.3. Comparison with Existing Standards and Proposed Clinical Guidance Ranges

Our Thai-specific targets for underweight (11.5–18.0 kg) align closely with IOM 2009 (12.5–18.0 kg) and Siriraj 2014 (10–18 kg) [1,15]. For normal weight women, our P75 extends to 18.0 kg, above both IOM 2009 (16 kg) and Siriraj 2014 (16 kg). For overweight and obesity class I, our targets are substantially higher than both prior sources, reflecting the critical methodological difference that we derived targets from women correctly classified as overweight (BMI 23.0–24.9 kg/m^2^) rather than from the WHO overweight category (BMI 25.0–29.9 kg/m^2^). For obesity class II+, our targets (5.8–12.1 kg) converge towards IOM 2009 (5.0–9.0 kg), consistent with the physiological rationale that women with severe obesity benefit from restricted total GWG.

Acknowledging that this study represents a single-centre derivation and that clinical guidelines should reflect triangulation of multiple evidence sources, we propose provisional clinical guidance ranges for Thai pregnant women pending multi-site external validation (Table 5). A conservative synthesis logic was applied: where our empirically derived P75 exceeds established sources, we adopt the more conservative figure from those sources, noting that it falls well within our empirically acceptable range. The rules governing this synthesis, including how lower bounds were rounded and how groups with no comparable external source or with a small healthy subgroup were handled, are specified in full in Section 2.9 so that the provisional ranges can be reproduced and revised as further Thai data accumulate. For normal weight women, 16 kg is proposed as the upper bound rather than our P75 of 18 kg; since 16 kg lies comfortably within our health outcomes zone, this choice does not violate our data, but appropriately defers to the convergent evidence of two established sources. Preliminary GWG estimates previously published in Thai-language nursing textbooks were based on WHO cut-points; the present study’s empirically grounded, Thai BMI-classified targets provide the evidence base for updating those estimates. The development of national population-specific GWG standards has international precedent: Brazil recently replaced IOM guidelines and the Atalah curve with a national GWG reference derived from 7086 Brazilian women, demonstrating that a single large-scale national dataset can anchor a formal guideline update [32]. Thailand is well positioned to follow this model: the combined evidence from Sunsaneevithayakul et al. [15], Thanaratsiriworakul et al. [16], and the present study, when pooled across >7000 Thai pregnancies and evaluated against multiple outcomes, provides a comparable evidential basis for a nationally convened guideline process. The study provides the empirical grounding to update these estimates using Thai BMI classification. The extended zone safety data confirm that Thai-specific upper bounds exceeding IOM 2009 are not only empirically derived, but clinically safe, consistent with evidence from Taiwanese women showing that strict IOM adherence increased small for gestational age risk [33], and supporting the principle that targets must reflect the actual outcome distribution of the population of interest [34]. We emphasize that these ranges are provisional pilot estimates derived from one centre. Their intended function at this stage is to inform BMI-appropriate counselling within the study setting and to provide the empirical basis and sample-size assumptions for a multi-center Thai validation study, not to serve as a de facto national standard in advance of that work.

### 4.4. Translating Provisional Targets into Graded Nursing Practice

Thai antenatal services are delivered across a wide resource gradient, from regional and tertiary centers with obstetric, dietetic, and laboratory support to sub-district health-promoting hospitals and community midwifery stations staffed by one or two nurse-midwives with a scale, a sphygmomanometer, and a paper record. A single uniform protocol is therefore not implementable, and recommendations pitched only at the level of courses and standardized charts risk being adopted where they are least needed. We propose instead a graded model with a common minimum core and a tier-dependent extension. Table 6 sets out the BMI-stratified surveillance schedule that constitutes the clinical content, and Table 7 distributes that content across two service tiers. The minimum core, feasible anywhere a scale exists, comprises four elements: weight measured and plotted at every antenatal contact; BMI classified at booking using Thai cut-points; a single BMI-appropriate target range communicated verbally and recorded in the woman’s hand-held book; and a defined referral trigger when the trajectory departs from that range on two consecutive visits. Tier 1 units add individualized dietetic assessment, structured group education, and, where available, digital trajectory monitoring of the kind shown to be effective in nurse-led Asian mHealth trials [31]. Tier 2 units are supported by a laminated one-page BMI-classified GWG chart, a scripted three-minute counselling sequence (classify, plot, agree one behavioral goal, set the next weighing date), and a single-page referral criterion sheet, all of which can be delivered without additional staffing or equipment. Evidence from routine-care implementation trials indicates that brief, structured, goal-directed counselling supported by a chart is the active ingredient in improving GWG adherence [19,30], which is precisely the component that transfers to low-resource settings.

## 5. Limitations

Several limitations of this study require consideration. First, this single-center study is not nationally representative. The targets reported here should be read as pilot estimates generated in one regional population, and internal validation using a 70:30 split of the same dataset does not substitute for external multi-site validation. We regard the principal contribution of this work to be that it demonstrates that Thai BMI classification materially changes GWG risk assessment, and that providing the effect sizes and distributional assumptions is needed to design an adequately powered multi-centre Thai study, rather than establishing ranges ready for national adoption. Second, GDM ascertainment was subject to a systematic protocol change between years: selective GCT-based screening in Years 2023 and 2024 versus universal OGTT in Year 2025. The sensitivity analyses reported in Section 3.1 indicate that much of the apparent secular rise is attributable to this change, and the trend should not be interpreted as a validated estimate of rising incidence. Some cases under selective screening in the earlier years will have been missed despite direct electronic record verification, which may also have introduced non-differential misclassification of GDM status into the regression models, biasing associations with GWG towards the null. Third, pre-pregnancy weight was based on the first antenatal visit weight (≤14 weeks), which may slightly overestimate true pre-pregnancy weight, particularly in heavier women. Fourth, the obesity class II+ healthy-outcomes subgroup comprised only 27 women. A 25th and 75th percentile estimated from 27 observations carries wide sampling uncertainty, and the derived range of 5.8–12.1 kg should be regarded as indicative of the direction of difference from other BMI groups rather than as a defensible clinical target in its own right. Until this group is characterized in a larger sample, we recommend that obesity class II+ women continue to be counselled using existing IOM 2009 guidance, with the provisional 5–12 kg range used only as supporting context and with monitoring for PE/GHT prioritized given the 16.1% event rate observed in this group. Fifth, the study did not capture data on dietary intake, physical activity, socioeconomic status, or gestational weight gain timing. These are established determinants of both GWG and the outcomes examined, and their absence is the most important constraint on the regression models: the adjusted odds ratios reported in Table 4 are only adjusted for maternal age and nulliparity and are therefore subject to substantial residual confounding. They should be interpreted as adjusted associations within this cohort and not as causal or independent effects of GWG. The likely direction of this confounding is not uniform: lower socioeconomic status is plausibly associated with both inadequate gain and adverse neonatal outcomes, which would inflate the observed associations for low birth weight and preterm birth, whereas dietary quality may operate in the opposite direction for GDM. so neither an inflated nor an attenuated overall bias can be assumed. Sixth, and relatedly, nursing-process variables were not available for the full cohort. Antenatal visit frequency, receipt of individual nutrition counselling, and the intensity of weight-related advice all plausibly lie on the causal pathway between BMI group and outcome, and their omission means that the portion of risk attributable to, or modifiable by, nursing care cannot be quantified from these data. Establishing that quantity is a specific objective we recommend for the prospective multi-center study proposed above.

## 6. Conclusions

This study presents the first Thai-specific gestational weight gain targets derived using Thai BMI cut-points across five BMI subgroups, validated using a healthy outcomes methodology and internal derivation/validation split. The targets are provisional, single-center pilot estimates. Within this cohort, they were not associated with excess risk relative to IOM-adequate gain and were substantially more achievable than IOM 2009 criteria (adherence 50.2% versus 34.7%). Furthermore, continuous dose–response modelling using restricted cubic splines confirmed that the lowest predicted risks for adverse outcomes aligned closely with these derived ranges. The critical finding that inadequate GWG in obesity class I women is associated with a nearly fourfold increase in GDM risk challenges the common practice of indiscriminate weight restriction counselling in obese women and underscores the importance of target-range-based, not ceiling-based, guidance. Triangulating across three evidence sources, provisional clinical guidance ranges of 11–18, 10–16, 9–16, 8–16, and 5–12 kg are proposed for underweight, normal weight, overweight, obesity class I, and obesity class II+ Thai women, respectively, pending external multi-site validation. For nursing and midwifery practice, four operational points follow directly and can be implemented at any level of service (Table 6 and Table 7): weigh and plot at every antenatal contact and classify BMI at booking using Thai rather than WHO cut-points; communicate one BMI-appropriate target range, with an explicit lower bound as well as an upper bound; screen women in the overweight, obesity class I, and obesity class II+ groups for glucose intolerance at the first antenatal visit with repeat testing at 24–28 weeks, and measure blood pressure at every visit with a lowered threshold for proteinuria testing in obesity class II+; and treat a trajectory that falls below the target range in a woman with obesity class I as a metabolic warning sign warranting earlier glucose assessment rather than as an occasion for further dietary restriction. These findings and the accompanying graded implementation model provide a defensible starting point for a multi-center Thai validation study, which we regard as a prerequisite to any revision of national antenatal care guidelines, and an immediately usable basis for incorporating population-specific GWG counselling into nursing and midwifery education.

## Figures and Tables

**Table 1:** Baseline characteristics of the study population by BMI group (*N* = 1396).

Characteristic	Total (*N* = 1396)	UW (*n* = 169)	NW (*n* = 644)	OW (*n* = 223)	OB1 (*n* = 267)	OB2 (*n* = 93)
Age (years), mean ± SD	29.5 ± 5.2	25.7 ± 5.0	29.4 ± 5.1	30.6 ± 5.1	30.6 ± 5.2	30.2 ± 5.5
Nulliparous, *n* (%)	762 (54.6)	120 (71.0)	373 (57.9)	111 (49.8)	121 (45.3)	37 (39.8)
Pre-preg BMI, mean ± SD	22.9 ± 4.2	17.3 ± 0.9	20.7 ± 1.2	23.9 ± 0.6	27.2 ± 1.4	32.7 ± 3.0
GWG (kg), mean ± SD	13.8 ± 5.7	14.9 ± 4.9	14.7 ± 5.2	14.2 ± 5.8	12.8 ± 6.5	10.0 ± 5.9
GDM, *n* (%)	220 (15.8)	19 (11.2)	89 (13.8)	39 (17.5)	48 (18.0)	25 (26.9)
PE/GHT, *n* (%)	46 (3.3)	3 (1.8)	9 (1.4)	11 (4.9)	8 (3.0)	15 (16.1)
Caesarean section, *n* (%)	450 (32.2)	30 (17.8)	184 (28.6)	80 (35.9)	118 (44.2)	38 (40.9)
LBW (<2500 g), *n* (%)	70 (5.0)	14 (8.3)	36 (5.6)	8 (3.6)	10 (3.7)	2 (2.2)
Macrosomia (≥4000 g), *n* (%)	35 (2.5)	1 (0.6)	14 (2.2)	3 (1.3)	12 (4.5)	5 (5.4)
Preterm birth (<37 wks), *n* (%)	75 (5.4)	11 (6.5)	35 (5.4)	12 (5.4)	12 (4.5)	5 (5.4)

Note: UW = underweight (<18.5 kg/m^2^); NW = normal weight (18.5–22.9 kg/m^2^); OW = overweight (23.0–24.9 kg/m^2^); OB1 = obesity class I (25.0–29.9 kg/m^2^); OB2 = obesity class II+ (≥30.0 kg/m^2^). GDM = gestational diabetes mellitus; PE/GHT = preeclampsia/gestational hypertension; LBW = low birth weight.

**Table 2:** Thai-specific GWG targets compared to IOM 2009 and Siriraj 2014 recommendations.

BMI Group (Thai Criteria)	*n* (%)	*n* Healthy	Thai-Specific Target (P25–P75)	IOM 2009	Siriraj 2014 *
Underweight (<18.5)	169 (12.1%)	109	11.5–18.0 kg	12.5–18.0 kg	10–18 kg
Normal weight (18.5–22.9)	644 (46.1%)	362	11.5–18.0 kg	11.5–16.0 kg	8–16 kg
Overweight (23.0–24.9)	223 (16.0%)	101	9.7–18.0 kg	7.0–11.5 kg ^†^	6–14 kg ^†^
Obesity class I (25.0–29.9)	267 (19.1%)	107	9.1–16.0 kg	5.0–9.0 kg ^†^	4–8 kg ^†^
Obesity class II+ (≥30.0)	93 (6.7%)	27 ^‡^	5.8–12.1 kg ^‡^	5.0–9.0 kg	4–8 kg

Note: * Siriraj 2014 used WHO cut points (OW ≥ 25.0 kg/m^2^); values for OW and OB1 rows are not directly comparable as they derive from a differently classified population. ^†^ Thai-specific target upper bound exceeds IOM/Siriraj for this group. ^‡^ Obesity class II+ healthy subgroup *n* = 27; interpret with caution.

**Table 3:** GWG classification under three criteria systems applied to the same 1396 women.

GWG Criteria	BMI System	Inadequate *n* (%)	Adequate *n* (%)	Excessive *n* (%)	Adherence
IOM 2009	WHO (OW ≥ 25.0)	361 (25.9%)	484 (34.7%)	551 (39.5%)	34.7%
Siriraj 2014	WHO (OW ≥ 25.0)	158 (11.3%)	729 (52.2%)	509 (36.5%)	52.2%
Thai-specific (present study)	Thai (OW ≥ 23.0)	366 (26.2%)	701 (50.2%)	329 (23.6%)	50.2%

Note: All three criteria applied to the same 1396-woman dataset. Thai-specific criteria use Thai BMI cut-points (OW ≥ 23.0 kg/m^2^); IOM 2009 and Siriraj 2014 use WHO cut-points (OW ≥ 25.0 kg/m^2^). Differences in the ‘inadequate’ proportions across systems reflect the different GWG lower bounds applied to the overweight group under each classification.

**Table 4:** Multivariable logistic regression for adverse pregnancy outcomes by GWG category within BMI group (significant findings, *p* < 0.05).

Outcome	BMI Group	GWG Category	*n*	Event Rate (%)	aOR (95% CI)	*p*-Value
Gestational diabetes mellitus	OB1 (25.0–29.9)	Inadequate	116	35.3	3.92 (2.12–7.24)	<0.001 *
	OW (23.0–24.9)	Excessive	60	6.7	0.29 (0.09–0.88)	0.029 *
Caesarean section	NW (18.5–22.9)	Inadequate	168	20.8	0.57 (0.36–0.90)	0.016 *
Preeclampsia/GHT	OW (23.0–24.9)	Excessive	60	11.7	7.57 (1.51–37.86)	0.014 *
Low birth weight	NW (18.5–22.9)	Inadequate	168	8.9	3.43 (1.46–8.08)	0.005 *
Preterm birth	NW (18.5–22.9)	Inadequate	168	7.7	2.83 (1.20–6.63)	0.017 *

Note: Adequate GWG (within Thai-specific P25–P75) is the reference category for all odds ratios. Event rate (%) is the outcome rate within that specific GWG-category subgroup and should not be compared directly across rows. The 35.3% GDM rate in OB1 inadequate reflects that women with obesity who fail to achieve adequate weight gain represent those with the most severe underlying metabolic profiles; this is consistent with inadequate GWG being a symptom of pre-existing insulin resistance in this group. aOR = adjusted odds ratio, adjusted for maternal age and nulliparity; CI = confidence interval; GDM = gestational diabetes mellitus; PE/GHT = preeclampsia/gestational hypertension; LBW = low birth weight; NW = normal weight; OW = overweight; OB1 = obesity class I. * *p* < 0.05. Obesity class II+ (*n* = 93) excluded due to insufficient cell frequencies for stable regression.

**Table 5:** Proposed provisional clinical guidance ranges for Thai pregnant women (pending multi-site validation).

BMI Group (Thai Criteria)	IOM 2009	Siriraj 2014 *	Present Study (P25–P75)	Proposed Provisional Clinical Range	Rationale
Underweight (<18.5 kg/m^2^)	12.5–18.0	10–18	11.5–18.0	11–18 kg	Lower: midpoint IOM/Siriraj; Upper: consistent across all three
Normal weight (18.5–22.9 kg/m^2^)	11.5–16.0	8–16	11.5–18.0	10–16 kg	Upper conservatively at 16 (IOM/Siriraj); within our P75; lower from IOM/present study
Overweight (23.0–24.9 kg/m^2^)	Not comparable ^†^	Not comparable ^†^	9.7–18.0	9–16 kg	Novel group; upper conservatively below P75; lower from P25
Obesity class I (25.0–29.9 kg/m^2^)	5.0–9.0 †	4–8 ^†^	9.1–16.0	8–16 kg	Present study dominates; IOM/Siriraj not applicable to this group
Obesity class II+ (≥30.0 kg/m^2^)	5.0–9.0	4–8	5.8–12.1 ^‡^	5–12 kg	Lower from IOM; upper from P75 (OB2 subgroup small, caution)

Note: * Siriraj 2014 used WHO cut points; OW/OB1 rows are not directly comparable. ^†^ IOM/Siriraj OW category (BMI 25.0–29.9 kg/m^2^) corresponds to OB1 under Thai classification; these are therefore not applicable to the Thai-defined OW group. ^‡^ *n* = 27 in the obesity class II+ healthy subgroup; interpret with caution. Prov. = provisional.

**Table 6:** Proposed BMI-stratified nursing surveillance schedule accompanying the provisional GWG ranges.

BMI Group (Thai Criteria)	Provisional GWG Range	Weight Monitoring	Glucose Screening	BP/PE Surveillance	Referral Trigger
Underweight (<18.5)	11–18 kg	Every visit; plot trajectory; review at 20, 28, 36 weeks	Routine screening at 24–28 weeks	BP every visit	Gain below range on two consecutive visits, or any weight loss
Normal weight (18.5–22.9)	10–16 kg	Every visit; plot trajectory	Routine screening at 24–28 weeks	BP every visit	Trajectory outside range on two consecutive visits
Overweight (23.0–24.9)	9–16 kg	Every visit; plot trajectory; reinforce counselling at every contact	Screen at first visit; repeat at 24–28 weeks if initially normal	BP every visit; urine protein if BP ≥ 140/90; heightened vigilance once GWG exceeds 18 kg	Cumulative GWG > 18 kg, or BP ≥ 140/90
Obesity class I (25.0–29.9)	8–16 kg	Every visit; plot trajectory; dietetic review if gain is below range	Screen at first visit; repeat at 24–28 weeks if initially normal	BP every visit; urine protein if BP ≥ 140/90	Gain below range (metabolic red flag warranting earlier glucose testing) or above 16 kg
Obesity class II+ (≥30.0)	5–12 kg	Every visit; plot trajectory; individualized dietetic input	Screen at first visit; repeat at 24–28 weeks if initially normal	BP every visit; assess eligibility for preeclampsia prophylaxis per national guidance; low threshold for proteinuria testing	Any BP ≥ 140/90, proteinuria, or GWG outside range

Note: Frequencies and thresholds follow current Thai national antenatal guidance [7] and are proposed, not validated; local adaptation is required. BP = blood pressure; PE = preeclampsia; GWG = gestational weight gain.

**Table 7:** Two-tier implementation of the surveillance schedule across Thai antenatal services.

Component	Tier 1: Regional/Tertiary Antenatal Unit	Tier 2: Community Midwifery Station or Sub-District Health-Promoting Hospital
Weight measurement and charting	Digital scale; weight auto entered to the electronic antenatal record with automated trajectory alerts	Mechanical scale; weight plotted by hand on a laminated one-page BMI-classified GWG chart kept in the hand-held record
BMI classification	Calculated automatically at booking using Thai cut-points	Single-page BMI look-up table using Thai cut-points, height measured once at booking
Counselling	Individualised dietetic assessment plus structured group education; motivational interviewing where staff are trained	Scripted three-minute sequence at every visit: classify, plot, agree one behavioral goal, set next weighing date
Educational materials	Full nutrition education package with locally costed meal examples; digital or mHealth trajectory monitoring where available [31]	Pictorial nutrition education script using locally available foods and portion sizes; no literacy or connectivity requirement
Glucose and BP surveillance	Per Table 6, with on-site laboratory and same-visit results	Per Table 6, with point-of-care glucose testing and scheduled referral for diagnostic testing
Referral pathway	Internal referral to obstetrician, dietitian or diabetes nurse specialist	Defined written referral criteria (Table 6) with a named receiving unit and transport plan
Staff preparation	Continuing education module with competency assessment	One-hour orientation to the chart and script; annual refresher

Note: Both tiers share the same minimum core: weight at every contact, Thai BMI classification at booking, one BMI-appropriate target range communicated and recorded, and a defined referral trigger. The tiers differ in the intensity of the extension, not in the underlying targets.

## Data Availability

The datasets generated and analyzed during the current study are available from https://cmu.to/qsd8g.

## Supplementary Materials

The following supporting information can be downloaded at https://www.mdpi.com/article/10.3390/nursrep16080274/s1. Table S1: Multivariable logistic regression model evaluating the association between calendar year and gestational diabetes mellitus (GDM), adjusted for screening protocol and maternal characteristics.

## Public Involvement Statement

No public involvement in any aspect of this research.

## Guidelines and Standards Statement

This study was prepared in accordance with the STROBE (Strengthening the Reporting of Observational Studies in Epidemiology) Statement, which provides guidelines for the reporting of observational studies.

## Abbreviations

The following abbreviations are used in this manuscript:

GWG	Gestational weight gain
BMI	Body mass index
GDM	Gestational diabetes mellitus
