# Supplementary material for: Development of Thai-Specific Gestational Weight Gain Targets Using Asian BMI Cut-Points: Implications for Nursing and Midwifery Practice in Preventing Gestational Diabetes Mellitus
Source: Nurs Rep. 2026 Aug 4;16(8):274. doi: 10.3390/nursrep16080274 (PMC13516527; doi:10.3390/nursrep16080274)
Supplement: Supplementary file 1 [file nursrep-16-00274-s001.zip › nursrep-4422984-supplementary.pdf]

Supplementary Materials

Development of Thai-Specific Gestational Weight Gain Targets Using Asian BMI Cut-Points: Implications for Nursing and Midwifery Practice in Preventing Gestational Diabetes Mellitus

Table S1. Multivariable logistic regression model evaluating the association between calendar year and gestational diabetes mellitus (GDM), adjusted for screening protocol and maternal characteristics.

Predictor	Adjusted Odds Ratio (aOR)	95% Confidence Interval	p-value
Calendar year (per year increase)	1.25	0.87–1.80	0.234
Screening protocol (Universal vs. Selective)	0.97	0.52–1.82	0.923
Maternal age (years)	1.13	1.10–1.17	<0.001*
Nulliparity (Yes vs. No)	1.37	0.99–1.87	0.054
**BMI category**			
Normal weight (Reference)	1	–	–
Underweight	1.04	0.61–1.80	0.879
Overweight	1.31	0.85–2.01	0.226
Obesity class I	1.27	0.86–1.89	0.228
Obesity class II+	1.85	1.09–3.13	0.022*

Note. Model estimated using maximum likelihood (N = 1,396). The association of calendar year with GDM prevalence was not statistically significant after controlling for the mid-study change in screening protocol and baseline maternal risk factors. * $p < 0.05$.
